# Mini Review: Deep Learning for Atrial Segmentation From Late Gadolinium-Enhanced MRIs

**DOI:** 10.3389/fcvm.2020.00086

**Published:** 2020-05-27

**Authors:** Kevin Jamart, Zhaohan Xiong, Gonzalo D. Maso Talou, Martin K. Stiles, Jichao Zhao

**Affiliations:** ^1^Auckland Bioengineering Institute, The University of Auckland, Auckland, New Zealand; ^2^Waikato Clinical School, Faculty of Medical and Health Sciences, The University of Auckland, Auckland, New Zealand

**Keywords:** atrial fibrillation, left atrium, machine learning, image segmentation, convolutional neural network, LGE-MRI

## Abstract

Segmentation and 3D reconstruction of the human atria is of crucial importance for precise diagnosis and treatment of atrial fibrillation, the most common cardiac arrhythmia. However, the current manual segmentation of the atria from medical images is a time-consuming, labor-intensive, and error-prone process. The recent emergence of artificial intelligence, particularly deep learning, provides an alternative solution to the traditional methods that fail to accurately segment atrial structures from clinical images. This has been illustrated during the recent 2018 Atrial Segmentation Challenge for which most of the challengers developed deep learning approaches for atrial segmentation, reaching high accuracy (>90% Dice score). However, as significant discrepancies exist between the approaches developed, many important questions remain unanswered, such as which deep learning architectures and methods to ensure reliability while achieving the best performance. In this paper, we conduct an in-depth review of the current state-of-the-art of deep learning approaches for atrial segmentation from late gadolinium-enhanced MRIs, and provide critical insights for overcoming the main hindrances faced in this task.

## Introduction

The ability to perform body imaging has been described as one of the most important revolutions in medicine of the past 1,000 years for its contribution to medical prevention, diagnosis, and prognosis (1). Since then, medical imaging has never ceased to improve, allowing cardiologists, and researchers to assess heart size using chest x-rays (2), to evaluate heart mechanical work with echocardiography imaging (3–5) and to accurately determine the heart's dimensions using cardiac magnetic resonance imaging (MRI) (6). Due to its good image quality, excellent soft-tissue contrast, and absence of ionizing radiation, MRI has become the gold standard modality to precisely identify patients' cardiac structures and etiology, guiding diagnosis and therapy decisions (7).

Improvements of MRI techniques, particularly with the aid of contrast agents such as gadolinium, led to the development of late gadolinium-enhanced MRI (LGE-MRI), allowing for the detection of scar tissue located within the myocardium. This technique has been extensively employed for clinical studies at Utah University (8–10) to analyze and understand the role of fibrosis and underlying structures that sustain atrial fibrillation (AF), the most common cardiac arrhythmia predicted to become a new epidemic in the coming decades (11, 12). They notably demonstrated the correlation between an increased amount of fibrosis present in the left atrial (LA) wall and a poor outcome of AF ablation (10). Over time, LGE-MRIs have become a widely accepted technique of choice allowing the detection and quantification of scar tissues located in the atrial wall.

**Figure 1:**
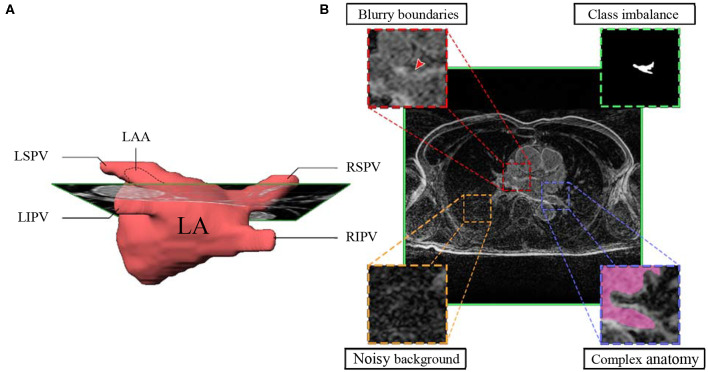
Main hindrances faced in LA segmentation from LGE-MRIs. **(A)** 3D representation of the complex anatomy of LA. **(B)** A typical 2D LGE-MRI extracted along the green rectangle from **A**), annotated with the main hindrances (blurry boundaries, class imbalance, noisy background, and complex anatomy) encountered in atrial segmentation. LGE-MRI, late gadolinium-enhanced magnetic resonance image; LA, left atrium; LAA, left atrial appendage; LSPV, left superior pulmonary vein; LIPV, left inferior pulmonary vein; RSPV, right superior pulmonary vein; RIPV, right inferior pulmonary vein.

The currently widely used clinical practice, including those conducted at Utah University, to analyze atrial structures and determine and quantify fibrosis distribution is by performing manual segmentation of the LA chamber from LGE-MRIs. However, the LA cavity represents a small volume (73 ± 14.9 cm^3^), constrained by a thin atrial wall (2–3 mm) and comprised of complex anatomy (13–15). Moreover, the anatomical structures surrounding the atria display similar intensities that can mislead some segmentation algorithms (16) (Figure 1). As a consequence, manual segmentation of the atrium is a time-consuming, labor-intensive, and error-prone process (8, 17, 18).

Before the advent of deep learning, researchers tried to develop and improve automated approaches to alleviate the burden of manual segmentation (19, 20). Earlier algorithms proposed would require important manual tunings such as thresholding methods or region growing approaches (21, 22). Other methods were later developed to provide a higher degree of automation using classifiers or clustering approaches such as *k*-nearest-neighbor (23) or *k*-means clustering (24), respectively. More recent methods, using statistical classifiers like support vector machine (25), active shape model (26), or multi-atlases (27) approaches, gained increasing interest for medical image analysis and cardiac segmentation. Though many of these approaches showed promising results, none presented enough consistency to be implemented widely in clinical practice.

In recent years, the development of more powerful computational hardware and the growth of clinical databases enabled deep learning, a subset of artificial intelligence (AI) (28–32) capable of automatic feature extraction and learning, to achieve tremendous advances notably in image classification and segmentation (33, 34). When applied to clinical images, deep learning even surpassed human-level accuracies for the detection of cancer on cervical images (35). Certain architectures employed for deep learning have also been proven to be very effective when applied to cardiac imaging. For example, Avendi et al. (36, 37) used a three-stage approach combining convolutional neural network (CNN), stacked encoder, and deformable models to segment the left ventricle (and later the right ventricle) on a small MRI dataset of 45 patients. On the other hand, Bai et al. (38) used a large MRI dataset provided by the UK Biobank database to develop their CNN for ventricular chamber assessment (volume, mass, ejection fraction) and segmentation, obtaining accuracy scores competing with human-level precision.

This increasing interest around deep learning can also be seen in the number of participants using deep learning approaches for the various challenges designed to promote the development of more robust methods for cardiac image segmentation (39–41). Atrial segmentation is becoming a matter of greater importance and can highly benefit from the development of deep learning. As an example, during the 2018 Atrial Segmentation Challenge, 15 of the 17 published approaches used deep learning to segment the LA cavity from LGE-MRI images, yielding high accuracy results and outperforming conventional segmentation approaches (42). The number is in sharp contrast with the previous atrial segmentation challenge held in 2013, during which only one approach used a learning algorithm (16). Thus, this growing interest for deep learning in research challenges illustrates the shift occurring in atrial segmentation and more broadly in clinical imaging development, moving more and more toward deep learning-based approaches that will revolutionize clinical practice in the coming years.

In this paper, we aim to provide an analysis of the current deep learning technique used for atrial segmentation on LGE-MRIs. Firstly, we will describe some of the fundamental concepts employed in deep learning for medical image segmentation. Subsequently, we will detail the various deep learning approaches addressing the main obstacles faced performing automated atrial segmentation. Finally, we will conclude our review with an outline of future developments for atrial segmentation using deep learning and more broadly the future of AI in clinical practice.

## Core Concepts of Deep Learning

Since Alan Turing published his article “Computing Machinery and Intelligence” asking “Can machines think?” researchers have thrived to comprehend, develop, and achieve AI (43, 44) although today, after over 60 years, general AI is still not within reach. Nevertheless, in recent years, the growth of computer processing power and technologies has allowed researchers to develop algorithms capable of learning proficiently through deep learning using artificial neural networks (ANNs). As ANNs represent the most popular structure to perform deep learning, this section will describe the core concepts of ANNs and their various practical use in medical imaging.

### Artificial Neural Networks

**Figure 2:**
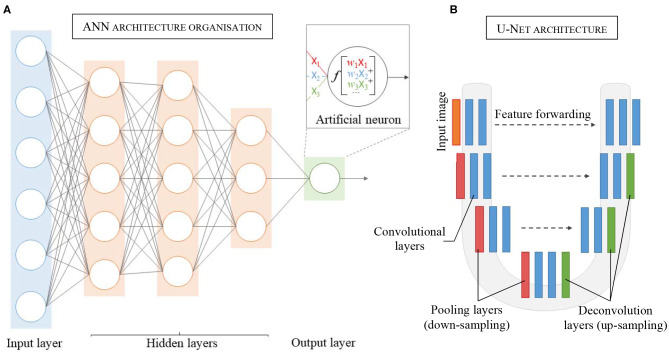
Schematic representation of the layered structure of an Artificial Neural Network (ANN), each circle representing an artificial neuron (details in the insert). **(A)** Each neuron receives inputs (*X*_1_, *X*_2_, *X*_3_), which are weighted (*w*_1_*X*_1_, *w*_2_*X*_2_, *w*_3_*X*_3_) and passed through an activation function *f*. **(B)** Architecture and details of one of the most popular convolutional neural network: U-Net.

Inspired by the biological neural networks found in the human brain (45), an ANN represents a collection of connected and tunable computational units, called artificial neurons, organized in a layered structure comprising a network (Figure 2A). Each neuron is a processing unit that can take multiple inputs. Each input is multiplied by an adjustable parameter called weight. All weighted inputs are summed together and passed through a non-linear function to yield a single output (30). Neural networks can address complex, highly non-linear problems due to the layered and connected structure of ANNs. In particular, the introduction of more advanced feature learning tools such as convolutional layers, the improvement of large datasets and better activation functions, e.g., ReLU, greatly helped the development of deep learning for segmentation tasks.

The key attribute of an ANN lies in its ability to learn the unique traits of a dataset by adjusting its weights accordingly during a training process. Typically, the weights are randomly initialized at the start of training. The training process can then be described in three consecutive phases: (1) forward propagation, (2) error calculation, and (3) back-propagation. In the forward propagation stage, the input data (e.g., LGE-MRI image) is fed to the network and flows through the different layers that extract the characteristic traits of the data, to ultimately yield a prediction (e.g., desired segmented image). The prediction is then compared to a reference data (e.g., manually segmented image by experts), called labeled data, and error is calculated using a dedicated function (called loss function). Finally, the weights are modified to minimize the estimated error, improving prediction accuracy. These three phases are repeated several times until the error converges to a significant minimized value.

### Different Tasks, Different Networks

Medical imaging encompasses a wide field of applications, and different tasks can represent different aspects of a diagnosis. Examples include the detection of an abnormal ECG signal, its classification as AF (46), or even atrial segmentation for planning for AF ablation (47). Therefore, each task requires a specific ANN architecture to properly model the desired operator, as the inputs and output can be drastically different depending on the nature of the task to be performed.

The number, types, and connections of layers in an ANN defines the network architecture. The CNN model is one of the most widely employed architectures in image analysis. CNN is a specific ANN architecture in which its hidden layers comprise one or more convolutional layers. The convolutional layers act as feature extractors from the input image, applying different convolution kernels to the initial image to generate feature maps containing meaningful information. Moreover, in convolutional layers, each artificial neuron receives their inputs from multiple neighboring neurons from the previous layer, sharing their weights and keeping the most spatially relevant information. This feature also allows a reduction in the number of parameters to adjust and therefore lowers the computational processing cost. Generally inserted in between sets of successive convolutional layers are pooling layers that are used to reduce the dimensionality of each generated feature map while retaining the relevant information. This down-sampling of the feature maps, typically by a factor of two, allows reduction of the computational cost while enlarging the field of view for the later convolutional layers.

For CNNs dedicated to image classification or detection, the architectures usually incorporate a fully connected layer as an end layer to summarize all information contained in the feature maps into a unique final prediction (output). Furthermore, CNNs can also be adapted for segmentation tasks by discarding the final fully connected layer and incorporating up-convolution layers in the network (35). These networks are called fully convolutional networks (FCNs). Up-convolution layers allow up-sampling of the feature maps to produce, *in fine*, output with the same size as the original input size (48). Thus, FCNs using up-convolution layers can perform pixel-wise prediction and therefore image segmentation.

First proposed by Long et al. (33) for semantic segmentation, the FCN architecture has been adapted and further extended for medical imaging notably with U-Net, a U-shape architecture (Figure 2B) developed for segmentation of histological images (48). By using skip-connections between down-sampled feature maps and up-sampled feature maps, the U-Net architecture allows features forwarding between the encoding part and the decoding part of the network, preventing singularities and achieving higher accuracy (49–51). After winning the ISBI cell tracking challenge in 2015, U-Net became the principal FCN architecture for medical imaging segmentation. Other studies further developed the U-shape architecture to use 3D images as input to render the spatial resolution of anatomical structures more accurately (52, 53).

## Atrial Segmentation Using Deep Learning

In this section, we provide a summary of the main difficulties encountered in atrial segmentation and the state-of-the-art deep learning approaches developed from LGE-MRIs to address them. To this regard, many of the methods reviewed were proposed for the MICCAI 2018 Atrial Segmentation Challenge which represented a cornerstone for the development of deep learning approaches for atrial segmentation from LGE-MRIs. Firstly, we will analyze the main methods employed to address class imbalance issues, a recurrent problem in segmentation of small structures such as the LA. Secondly, we will review the approaches developed to exploit image context providing more information for semantic segmentation of the LA using multi-scale strategies. Next, we will analyze the impact of loss function selection regarding either volumetric segmentation or surface segmentation. Finally, we will discuss the influence of the input dimensionality (2D/3D) for atrial segmentation when dataset size represents a significant shortcoming.

### Multi-Stage CNN and Class Imbalance

**Figure 3:**
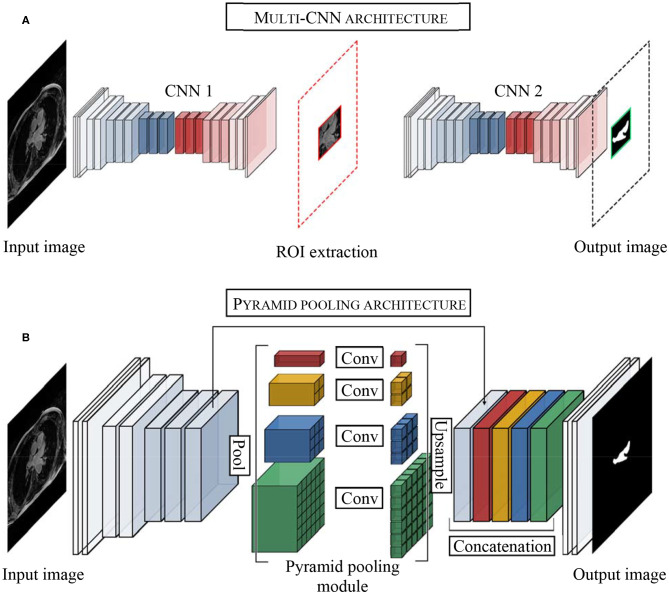
Examples of top architectures developed for atrial segmentation from LGE-MRIs. **(A)** Multi-stage CNN architecture uses the first convolutional neural network (CNN 1) to extract the region of interest (ROI) and the second convolution neural network (CNN 2) to perform the segmentation of the left atrium. **(B)** Pyramid pooling architecture increases contextual information in the learning process. Pool, pooling layer; Conv, convolutional layer.

One of the difficulties of atrial segmentation is that the atrial cavity represents only a small fraction of the image volume (~0.7%) and therefore creates a severe class imbalance between the over-represented background and the under-represented atrial structures, impairing the learning process. To address this issue, Vesal et al. (54) proposed to crop the input images from the center of the image, using fixed coordinates, to substantially remove the predominant background surrounding the LA. As a result, the learning process was entirely focused on a smaller region of interest (ROI), allowing better representation of the LA features. Based on a similar principle, other researchers (55–57), pushed this idea a step further by using a multi-CNN approach for atrial segmentation (Figure 3A). In their approaches, two consecutive networks were employed instead. The first CNN was specially trained to localize the LA on each input, allowing to subsequently crop out the unwanted background around the LA, as a prior step to segmentation. Then, the second network was dedicated to the segmentation task itself, focusing entirely on a small patch of each image.

Despite following a similar idea, it is important to distinguish these two methods. As the LA can show different positions on LGE-MRIs, using fixed coordinates from the center of the image to crop may result in unwanted cropping of relevant LA pixels. On the other hand, by dynamically centering the ROI on the LA for each input, multi-CNN approaches ensured the conservation of the atrial structures, cropping exclusively superfluous background pixels, and consequently optimizing background isotropy for the learning process.

To quantify the impact of each cropping approach, our recent study has investigated the importance of cropping the input patch to the CNN either from the center of the image (image-centered) or from the center of the LA (center of mass/centroid of the atrium) using different patch sizes (ranging from 240 × 240 to 576 × 576) (58). When using center cropping of the image, we did not observe any significant influence of the patch size on the Dice score (92.03 vs. 91.95% Dice score for 240 × 240 and 512 × 512 image size, respectively). On the other hand, cropping the images from the centroid of the LA using dynamic cropping, we noticed a significant increase in the accuracy when using small patches (240 × 240) compared to large patches (576 × 576) (Dice score 92.86 vs. 92.26%, *p* < 0.01). The utilization of LA centroid-centered patches allows the CNN to process a more condensed region of the large LGE-MRI scan as the exact location of the LA is known, reducing the class imbalance of each patch processed by the network.

### Multi-Scale Approaches and Context Learning

Another problem that decreases segmentation performance and limits the extraction of relevant cues during the training phase is the inconsistency in the sizes of the LA anatomical structures such as the pulmonary veins or the left atrial appendage seen in LGE-MRIs from different patients.

He et al. (59) initially developed a pyramid pooling module, a multi-scale pooling, intended to prevent object misclassification by using image context information. By incorporating multi-scale pooling, the CNN could associate contextual features, delivering more accurate classification. Based on this idea, Zhao et al. (60) proposed PSPNet, a neural network with pyramid pooling which incorporates object and image context to the learning process. These two approaches were developed using large miscellaneous datasets such as ImageNet (61), PASCAL VOC 2012 (62), or ADE20K dataset (63), and the pyramid pooling exploiting the context variability of the dataset, allowed to alleviate object miss-classification or segmentation errors.

Inspired by He et al. (59) and the PSPNet developed by Zhao et al. (60), Bian et al. (64) proposed a multi-scale 2D CNN using spatial pyramid pooling to extract different scale features of the training dataset (Figure 3B). Thus, by means of different pooling kernel sizes and their combination, they proposed a CNN able to learn different cue size and improve network robustness against high shape variability usually encountered in clinical datasets.

However, the dataset employed for this approach (154 3D LGE-MRIs of the chest cavity) does not provide as much contextual variability as the large image database aforementioned, but rather displays the same object (the LA) in the same anatomical context (the thoracic cavity), providing only a few contextual variations to train on. Thus, arguably using pyramid pooling module for LA segmentation in the chest cavity might only show limited benefits from context learning.

Pyramid pooling also grants the ability to generate a fixed-length vector on a fully connected layer for classification tasks. This was illustrated by Chen et al. (65) using the pyramid pool module to extract more information from the dataset and classify the images between pre-/post-surgery, as they used a deeper U-Net to segment the LA simultaneously.

Based on the similar idea of incorporating multi-scale cues during the learning process, Vesal et al. (54) employed dilated convolution layers (also called atrous convolution layers) at the deepest level of their network. These convolution layers use dilatation rates to enlarge their receptive fields, allowing the network to learn different scale features (66). However, at each convolution the receptive field of each neuron is increased, therefore if not used wisely, receptive fields can become larger than the input image, resulting in a waste of memory while not improving the learning process.

These approaches ensure the incorporation of shallow features (spatial cues) and deep features (semantic cues) during the learning process. Therefore, combining effective class imbalance management with contextual cues could potentially improve even more the current methods. However, cropping to the smallest ROI possible using a first CNN of a two-stage approach, like Xia et al. drastically reduces the image context shown to the network. Therefore, the pyramid pooling module might not be able to provide contextual cues from the LA surrounding structures to improve the learning process. Moreover, during the cropping process, the input image size is significantly reduced. Thus, the use of dilated convolution for segmentation in the second network of this strategy becomes almost obsolete as the receptive fields would quickly grow larger than the input image during the learning process. Thus, fusing these strategies, although interesting, needs to be considered wisely.

### Loss Function

The current main evaluation metrics employed in segmentation task using deep learning is the Dice score, for which a higher accuracy reflects almost exclusively a volume of pixel accurately annotated rather than well-defined anatomy. Hence, most of the deep learning approaches for segmentation employ pixel-wise segmentation relying either on cross-entropy loss function or dice loss function. However, these loss functions weigh more volume over contours, which can impair the learning of accurate boundaries in favor of a correct volume.

To improve boundary accuracy, several teams have developed contour-oriented loss functions. For example, Jia et al. (67) proposed a contour loss function (based on the pixel Euclidean distance) that decreases when the contour gets nearer to the reference contours of the label images during training, providing spatial distance information to the learning process. In their approach, they associated the dice function loss to obtain pixel-wise information, and their contour loss function for spatial information, achieving good shape consistency. In another strategy, Yang et al. (57) also defined a composite loss function, combining the overlap loss function (to reduce intersection between foreground and background) and a novel loss function called “focal positive loss” to guide the learning of voxel specific threshold and emphasize the foreground, improving, *in fine*, classification sensitivity. By recognizing ambiguous boundary location and enforcing positive prediction, this novel loss function improved the learning process and consequently the final atrial segmentation. However, these approaches did not obtain a better score then other approaches using more conventional loss function (e.g., dice loss, cross-entropy loss).

Therefore, it would be interesting to investigate the impact of a combined loss function allowing the network to learn from the volume (cross-entropy loss function or dice loss function) and from the contours of the LA. As segmentation tasks not only rely on minimizing volume error but also relies on boundaries accuracy (particularly for small structures). it is crucial to consider these two major aspects to ensure the reliability of the approach employed.

### Spatial Context (2D vs. 3D)

Even if clinical datasets are becoming bigger and better with the creation of centralized databases, for example, the UK Biobank (with more than 90000 3D MRI scans) (68), most of the current clinical databases available remain of humble size, making it difficult for a CNN to provide robust generalized solutions for segmentation. As an example, the current largest LGE-MRI dataset with only 154 3D LGE-MRIs (which represent nearly 9,000 2D images for training) appears relatively small when compared to the hundreds of thousands of images used for the major classification challenges for which the proposed approaches reach outstanding accuracy (59, 69, 70).

Thus, in this race of performance, it is important to consider how to make the best of the dataset employed. To this regard, the choice of the image dimensions employed (2D and 3D) approaches must be considered wisely. As 2D approaches need considerably fewer trainable parameters to yield good results, they are less gluttonous regarding memory consumption, and therefore require less time during the training process. Moreover, 2D approaches allow the processing of bigger batches of images compared to 3D approaches, as they require less memory to be processed. Therefore, 2D methods, using bigger batch size, help reduce gradient fluctuation and lead to faster convergence during the learning process. Additionally, 2D approaches can exploit more efficiently small datasets, reducing the risk of overfitting as the neural networks are fed with more images for the learning.

On the other hand, 3D approaches provide better spatial representation, fully exploiting data dimensionality as well as inter-slice continuity during training. This allows the network to learn major spatial features to render a more accurate 3D anatomy and yield, *in fine*, higher accuracy. Moreover, with the ever improvement of GPU technology, the current memory limitations will become of less importance in the near future; therefore, 3D approaches will become easier to use. Furthermore, as datasets are growing better and bigger, 3D approaches will be able to rely on more data and become more and more prominent in clinical imaging deep learning.

Nevertheless, relying on 2D images, Puybareau et al. (71) tried to improve the spatial representation of their dataset using a method called “pseudo-3D.” Their method employed the generation of color images from the 2D grayscale images, each slice being color expanded into the R, G, B space using slice *n-1*, slice *n* and slice *n*+*1*, to generate a three-channel image. This approach allows an improved spatial representation and alleviates low contrast intensity between atrial tissues and background and enrich the dataset. However, even if this approach does not provide the expected spatial representation, it can be a method of choice if resources are limited.

Following the multi-view approach developed by Mortazi et al. (72), Chen et al. investigated the possibility to combine 2D images and 3D representation (73). In their study, Chen et al. extracted the 2D images for each anatomical view (axial, coronal, and sagittal) from 100 3D LGE-MRIs. Then, they combined a first encoder-decoder network using long short term memory convolutional layers to preserve inter-slice correlation using the axial view, and a second network to learn complementary information from the sagittal and coronal views. Finally, the outputs for each view of the network were fused to yield LA and PV segmentation simultaneously. Using their approach, they obtained 90.83% Dice score accuracy for PV and atrial segmentation. Employing the same method, Yang et al. studied the influence of dilated convolution to counter image resolution variability encountered using a multi-view approach (74). Using 100 3D LGE-MRIs, they achieved 89.7% Dice score accuracy underlining the necessity to investigate systematic parameters tuning to obtain optimal performances on a task-specific basis.

In the present context, it is important to consider the trade-off using either a 2D approach requiring less memory and profiting more from the dataset (8,800 images rather than 154 3D LGE-MRIs) a 3D approach allowing more accurate spatial representation at the cost of longer and more difficult training. However, at the current stage, it is difficult to assess which method yields systematically better results. For example, during the 2018 Atrial Segmentation Challenge, the performances of 2D and 3D approaches remained very close (Table 1). Another possibility is to use a multi-view approach combining 2D images from different views to improve the spatial representation. These methods require training each view separately before combining the different output for the final prediction. While interesting, these methods still need improvement to reach the current state-of-the-art for atrial segmentation. Therefore, further improvements need to be sought regarding the size of the dataset, the number of approaches compared and the metrics employed to be able to draw a better conclusion.

**Table 1:** Summary of deep learning approaches developed for the 2018 atrial segmentation challenge.

**First Author**	**Summary**	**DC**	**Architecture**	**Pros/Cons**
Xia et al. (56)	2 stage network (LA localization, LA segmentation), Dice loss	93.2	2x 3D U-Net	Good class imbalance management, highest performance/computationally expensive
Bian et al. (64)	LA segmentation using, ResNet101, atrous convolutional layers and pyramid pooling, online hard negative example mining (objective function)	92.6	2D Pyramid Network	Multi-scale representation/competitive training can reinforce overfitting
Vesal et al. (54)	LA segmentation using manual cropping, dilated convolution at the deepest level of U-Net, combination of Dice loss and cross-entropy loss function	92.6	3D U-Net	Class imbalance management, new loss function/Risk of loss of information using center cropping
Li et al. (55)	2 stage network: 3D U-Net for detection, Hierarchical Aggregation network (HAANet) for LA segmentation, Dice loss	92.3	3D U-Net + HAANet	Class imbalance management/Slow and small benefits from Hierarchical mechanism (0.4%)
Puybareau et al. (71)	Assembly of three 2D gray-scale images to create RGB 2D color image, transfer learning (VGG), multinomial loss function for LA segmentation	92.3	VGG-Net	Fast to train, pseudo-spatial representation/pseudo spatial representation not multi-view or 3D
Yang et al. (57)	2 stage approach: LA detection (Faster-RCNN), LA segmentation (U-Net). Deep supervision, transfer learning. Composite loss function: Overlap loss and Focal Positive loss	92.3	Faster-RCNN/3D U-Net	Good ROI detection, composite loss function /Recursive training with risk of overfitting
Chen et al. (73)	LA segmentation and classification (pre/post-ablation) of images, using cross-entropy and sigmoid loss function, respectively	92.1	2D U-Net	Fast to train (2D), interesting data augmentation
Jia et al. (67)	2 stage network (LA localization, LA segmentation), contour loss	90.7	3D U-Net	Contour loss/computationally expensive
Liu et al. (75)	Manual center cropping, evaluation of 2 different networks U-Net and FCN for LA segmentation, Dice loss	90.3	2D U-Net and FCN	Quick (2D)/Native Unet/FCN
Borra et al. (76)	Otsu's algorithm for cropping, LA and pulmonary veins joined segmentation, Dice loss	89.8	3D U-Net	Otsu's for cropping/computationally expensive
de Vente et al. (77)	U-net for LA segmentation, Dice loss	89.7	2D U-Net	Fast (2D)/Native Unet
Preetha et al. (78)	Deep supervision (79) and U-Net for LA segmentation	88.8	2D U-Net	Deep supervision, Fast (2D)/Native Unet
Qiao et al. (80)	Multi-atlas selection and registration for LA segmentation	86.2	Multi-atlas	Groupwise registration/Slow prediction(multi-atlas)
Nuñez-Garcia et al. (81)	Multi-atlas whole heart labeling and shape-based atlas selection	85.9	Multi-atlas	Registration using gobal-atlases, shape based clustering/Difficulties do manage high variability in small dataset
Savioli et al. (82)	LA segmentation using V-Net and combination of mean squared error and Dice loss	85.1	3D V-Net	Composite loss function/computationally expensive

*DC, Dice Score; LA, Left Atrium*.

#### Evaluation Metrics

Another crucial point is to use metrics that provide a reliable evaluation of the final output using deep learning. One of the main scores employed is called Dice score and gauges the pixel-wise similarity between the predicted segmentation and the reference data. Dice score provides a good representation of the specificity and the sensitivity of the model. However, Dice score metric has some limitations as it only evaluates a percentage of pixel accurately annotated neglecting contours and shapes of organs that can be a critical part of diagnosis in clinical practice. Other metrics providing distance measurements, such as mean surface distance and Hausdorff maximum distance, are usually employed to provide an alternative evaluation. Mean surface distance estimates the average error (in mm) between the outer surfaces of the reference data and the predicted segmentation. Given the size and structure of LA, mean surface distance is a meaningful tool to reliably assess the anatomical boundaries of the predicted segmentation compared to the reference data. Hausdorff maximum distance (in mm) represents the maximum error between the surface of the predicted segmentation and the surface of the reference data. Therefore, Hausdorff distance indicates solely the distance at the worst part of the segmentation, providing only partial information of the correctness of the predicted segmentation. By combining mean surface distance and Hausdorff distance, it is possible to evaluate the fidelity of the boundaries of the segmented structures reliably. Finally, a more clinical aspect of the predictions can be examined to express the reliability of the approach by calculating volume error or anteroposterior atrial diameter error when comparing the segmented prediction with the reference image.

### Atrial Wall and Scar Segmentation

While the deep learning methods for atrial cavity segmentation on LGE-MRIs are effective, the more clinically relevant tasks, such as LA wall and fibrosis (scar) segmentation, remain challenging. For LA wall segmentation, several approaches have been developed using traditional strategies such as multi-atlas segmentation or graph-cuts method (83, 84). However, currently no deep learning approaches have been proposed for direct LA wall segmentation from LGE-MRI. Yang et al. (85) proposed a hybrid approach combining multi-atlases and an unsupervised sparse auto-encoders for LA scar segmentation. A multi-atlas algorithm was used to segment the LA blood pool from the LGE-MRIs. Then, this initial LA cavity segmentation was dilated uniformly by 3 mm to include the LA wall. Next, they used a sparse auto-encoder to delineate and segment the fibrosis from the atrial wall. They achieved 90 ± 0.12% Dice score for blood pool segmentation and 78 ± 0.08% Dice score for fibrosis segmentation. In their subsequent study (86), by fine-tuning the sparse auto-encoder parameters, the accuracy was improved to 82 ± 0.05% Dice score for fibrosis segmentation. While showing promising results, with these methods being only developed and tested on 20 3D LGE MRIs, they remain untested on larger datasets to assess their reliability against a broader range of anatomical variabilities regarding LA structures and fibrosis. Chen et al. (73) developed a CNN with an attention mechanism (87) to highlight salient features (in this case, the enhanced pixels of the scar tissues on LGE MRIs) and to force the model to focus on the scars locations. With this approach, Chen et al. obtained 77.64% Dice score for atrial scar segmentation using 100 3D LGE MRIs. This lower score (compared to that obtained from LA cavity segmentation) is potentially due to the scarcity of the LA scar pixels, which are small patches of inhomogeneous enhanced pixels within the atrial wall, impairing the extraction of meaningful features for fibrosis identification during the learning process of the CNN.

While these methods require atrial wall segmentation to be performed before fibrosis detection, Li et al. proposed a hybrid approach using a graph-cuts framework combined with a multi-scale CNN approach for direct scar identification (88). In their approach, the LA and PV were initially delineated using a multi-atlas segmentation method. Then fibrosis was segmented and quantified using a graph-cut network in which two neural networks were dedicated to predicting edge weights. The first network was dedicated to predicting the probabilities of a node belonging to scar or normal tissue, while the second network was devoted to evaluate the connection between two nodes, yielding, in fine, the fibrosis segmentation. By embedding the CNN networks in the graph-cut framework, Li et al. obtained a mean Dice score of 70.2% for scar tissue segmentation, showing the possibility of effectively assessing LA fibrosis without the need for prior wall segmentation. Thus, even if the two networks employed did not directly perform the fibrosis segmentation task, the CNNs contributed to the optimisation process refining the graph-cut approach used in this study. However, these methods tended to find fibrotic tissue out of the atrial wall boundaries regions, resulting in a drastic decrease in the final scores. Hence, the current models remain insufficient to provide anatomically accurate assessments allowing reliable fibrosis quantification due to the low Dice scores obtained. Thus, these approaches still require improvements to reach reliability and clinical applicability.

## Discussion and Conclusion

In this paper, we provided an in-depth analysis of the main automatic approaches using deep learning for atrial cavity segmentation from LGE-MRIs. Most of the proposed deep learning approaches for atrial segmentation used FCNs, most notably the very popular U-Net architecture. While U-Net is widely used for medical image segmentation in many disciplines (38, 89, 90), the discrepancy in the accuracy obtained between different studies still presents inherent issues involved in the generalized implementation of such architectures. By presenting a normalized survey of U-Net for the task of atrial segmentation, we showed the importance of proper class imbalance management, appropriate features extraction process, and meaningful loss function selection to yield precise and accurate atrial segmentation.

The current leading approach for LA segmentation from LGE-MRIs dataset involved a two-stage 3D CNN method which reached a remarkable Dice accuracy of 93.2%, currently the best-benchmarked performance using 100 3D LGE MRIs (42). In this approach, the first network reduces class imbalance effectively while optimizing background isotropy using dynamic cropping, providing the second network with a targeted region for more localized segmentation. Additionally, they employed extensive data augmentation to enhance the generalization capability of their approach. Finally, they employed a 3D approach reinforcing the features' spatial representation, allowing them to obtain the current highest score for LA segmentation using machine learning.

Small training datasets represent one of the main limitations of clinical datasets as annotation and data gathering remains difficult. For example, the current largest LGE-MRIs dataset only contains 154 cases and therefore cannot effectively represent human anatomical variability. In fact, in order to improve performance, most of the developed approaches rely heavily on data augmentation such as affine transformations, cropping and scaling to virtually enlarge the dataset, also taking the risk of introducing more artifacts in the dataset. Moreover, the annotation process of anatomical structures is a complex and tedious process, which can be seen in the inter/intra-observer variability reported in several studies (38, 91). For example, atrial structures such as the mitral valve are difficult to segment due to the lack of clear anatomical border between LA and left ventricle. Moreover, the PVs are a very thin structure and represent a challenge for experts to distinguish from other structures on poorly contrasted images, and current protocols for defining the degree of extension of the PVs from the LA wall still remains subjective. Thus, this labeling uncertainty leads to some label variability in the dataset used, impairing the training process and potentially misleading the deep learning algorithm for the prediction process. However, despite all these difficulties the study shows the success of deep learning approaches reaching a high Dice score accuracy (>90% Dice score), showing the importance of careful parameter selection and architecture design for achieving the best performance (38).

In this study, we showed the potential of applying deep learning to perform automatic segmentation of the LA directly from clinical imaging data. The current accuracy of the various approaches presented is promising for future clinical implementation by providing highly accurate anatomical maps of the LA. Additionally, multiple teams already proposed auspicious solutions for fibrosis assessment using deep learning, providing particularly valuable information for AF ablation strategies that could highly benefit initial patient stratification, diagnosis, prognosis, and potential guidance for an optimized ablation strategy. Moreover, the ability to generate high fidelity segmentations such as the LA opens the way for further applications of deep learning to segment other anatomical structures. For instance, high accuracy left atrial appendage segmentation would provide crucial information for atrial thrombosis risk assessment (92). Thus, practitioners would be able to provide adapted treatment strategies on time, potentially reducing the number of stroke accidents caused by migrating atrial thrombus. Additionally, LA segmentation approaches could also be applied to the RA, providing a better understanding of the role of fibrotic extents spread through the RA myocardium notably in sinoatrial diseases (93).

Finally, it is important to underline the limitation of the current metrics employed. As most of the segmentation tasks rely on pixel-wise classification, Dice score proposes an efficient way to determine the correctness of the overlapping prediction. However, Dice score can be defined as a volumetric metric as it weighs more generously toward an accurate volume over precise anatomical delimitations. In clinical practice, Dice score and volume accuracy are important for assessing LA dilatation, but becomes irrelevant when assessing boundaries of fine structures such as LA. Therefore, other metrics such as mean surface distance representing the distance between the labeled surface and the predicted surface should be considered to produce better anatomical accuracy evaluation. The Hausdorff distance, representing the maximum distance between two surfaces, can also be used to evaluate the maximum error between prediction and label, potentially guiding algorithms to minimize their maximum error. Moreover, other limitations such as variations in image quality and resolution or the introduction of image artifacts intrinsic to scanner manufacturer have to be taken to account for future clinical deployment. At the current stage, no study has investigated the influence of LGE-MRI image quality on the Dice score but empirically, the best image quality tends to yield higher accuracy scores. However, in clinical practice image quality can vary tremendously as cardiac motion, body fat, and chest breathing motion, amongst others, can generate artifacts to various degrees on the final images. Therefore, to provide good generalization capacity, deep learning models have to be able to extract meaningful features regardless of the quality of the image. Similarly to the image quality issue, to obtain good generalization capacity, a network should be trained with many images from many different scanners. Thus, large multi-center datasets need to be built to ensure satisfying scanner variability and image quality variability representation for the learning process. Finally, it is crucial to promote deep models with efficient inherent generalization capabilities, as different image resolutions can represent a major difficulty for deep learning models using large scale datasets. However, promising results were demonstrated using pyramid pooling architecture ensuring extraction of multi-scale features. Thus, at the current stage efforts remain to be made to develop a deep learning model satisfying these criteria for further clinical deployment.

With the development of computational hardware and the general effort to enrich medical image databases, the effectiveness of deep learning will only improve with time. Arguably, the current trend would lead to improve all fields of clinical practices as AI technologies become more widely developed and implemented. Furthermore, the current flourishing of the deep learning approaches in all areas of medical practice has already breached out research. Despite initial professional reluctance, AI technologies will become of major importance in the near future.

## Author Contributions

KJ and JZ conceived and designed the work. KJ searched and read the literature and drafted the manuscript. ZX, GM, MS, and JZ provided guidelines, critical revision, and insightful comments to improve the manuscript. All authors read and approved the manuscript.

## Conflict of Interest

The authors declare that the research was conducted in the absence of any commercial or financial relationships that could be construed as a potential conflict of interest.
